# Dr. Lubbock's Case of Catalepsy

**Published:** 1802-02

**Authors:** R. Lubbock

**Affiliations:** Norwich


GENTLEMEN,

THE following detail of a caſe of Catalepſy, which fell under my care a few years ago, may not be unworthy of a place in your uſeful Journal; and I ſhall alſo trouble you with two caſes of Diabetes. I am, &c.

*Norwich, Dec.* 10, 1802. R. LUBBOCK.

---

J. B. ætat. 23, a ſhopkeeper by trade, after experiencing ſome ſlight depreſſion of mind, and without any ſtrongly marked bodily weakneſs, was ſeized with ſlight and very ſhort attacks of inſenſibility; at firſt theſe attacks were not frequent, happening at uncertain periods, or perhaps only once in two or three days. In a ſhort time, however, they became more frequent, and he was ſeized ſeveral times in the day, ſo as to diſable him, in a great degree, for the execution of his duties as a ſhopman. At times he would be attacked when attending to his employment in the ſhop, and it made no difference whether he was in an erect or ſitting poſture, as he preſerved the ſame attitude until he regained his ſenſibility. At other times he would be taken during his meals, ſitting in the poſition in which the acceſſion aſſailed him, for ſome minutes, and carrying more the appearance of a ſtatue than of a living being.

In conſequence of the frequency of theſe attacks his employer wiſhed him to go home to his friends, and to follow ſome medical advice. In this ſituation I was deſired to ſee him, and found him contending with acceſſions that appeared to me truly of a cataleptic nature; they occurred frequently in the day, and equally whether he was in a ſitting, an erect, or reclined poſture. At one time, if he walked with his father a few hundred yards, he would be frequently ſtopped by an attack, when he would remain for a minute or two in an erect poſture, and ſtanding firmly upon his legs. And in my viſits I have occaſionally ſeen him in bed, and reclined, and upon the ſtretching out of either arm for the attainment of any object, he would be ſuddenly ſeized, and remain in a ſtate of inſenſibility for ſome time, with the arm ſtretched out, and forming a conſiderable angle with the trunk of the body. But the moſt extraordinary occurrence of this ſingular diſeaſe took place when he was viſiting ſome friends a few miles from his father's. One day having it only in contemplation to write a letter to his father, and without being in the act of writing, he was fuddenly feized with a fit; and fome by-ftanders obferving a motion in his right hand and fingers, refembling that of a perfon in the act of writing, a pen with ink was placed between the fingers, and fome paper being alfo conveniently placed, he regularly began a letter to his father, of which he wrote two or three lines, containing the common and ufual expreffions of duty, and faying that he hoped he fhould regain his health. The lines fo written were fhewn to me; and one day, during my vifit, obferving a fimilar motion of the hand, I witneffed the fame phenomenon of his writing about two lines of a fimilar letter.

In addition to the above mentioned particulars of this interefting malady, I wifh to fay that it continued for five or fix weeks, during which time volatiles, ether, the fœtid gums, opium, fome dofes of calomel, with the preparations of zinc and copper, were tried in vain; and medicine feemed to have little or no influence in reftraining the paroxyfms, until he took half a drachm of cinchona and the fame quantity of valerian in fubftance, every four hours, when they gradually decreafed, and left him entirely, and in the enjoyment of health. This patient had a fimilar attack fome months afterwards, although in a flighter degree, and which foon yielded to the fame medicines.

While I am fpeaking upon the fubject of Catalepfy, I wifh to add, that a few years ago I was called to vifit a young gentleman, of a delicate conftitution, labouring under a very fingular acceffion, and which I could clafs under no other head than that of Catalepfy. This patient, without any adequate or affignable caufe, would fall into a violent ftate of laughter, which would continue, to the fevere diftrefs of his friends, who were apprehenfive he might expire from fatigue, for fix or eight hours without remiffion, and in which ftate he feemed folely attentive to the bufinefs of laughing, being regardlefs of the obfervations, the wifhes, or the commands of thofe around him. This attack, I am aware, may by fome be deemed hyfterical, but from the torpor of mind to every other particular but the act of laughing, I feel difpofed to think it fomething cataleptic. This patient, after fome time, regained his health from a ftrengthening plan of medicine.

In a former Number of your Journal, I publifhed fome remarks upon the nature of Diabetes Mellitus; the treatment of which difeafe, although conducted upon the moft approved method, appeared to be unfuccefsful. I beg now to fay, that two of the three patients mentioned have been dead for feveral months; and from the ftate in which the third remained when

I heard

I heard of him, through a medical friend, some months ago, it is probable he is no more. In these cases it appeared that the liquid ingesta and the urinary discharge bore a more direct ratio to each other than has been usually supposed by authors, and that the reduction of the quantity of urine followed that of the liquid taken. The same remarks I have found to apply to the following cases, of which I have given a general outline; the one a case of Diabetes Mellitus, the other of Diabetes Insipidus.

John Barker, ætat. 38, a husbandman, was admitted into the Norfolk and Norwich hospital, September 12th, and was found to pass nearly eleven pints of pale straw-coloured urine in twenty-four hours, sweet to the taste, and a pint of which, by evaporation, yielded about half an ounce of saccharine extractive matter. Upon keeping an accurate account of the liquid taken and urine discharged, it appeared, that after the first few days, they were in a near proportion to each other, the urine not exceeding the liquid taken; so that if he took four, five, or six pints of liquid in twenty-four hours, rather less urine was generally passed. This patient was permitted to drink beer or any common liquid, and to take also the usual diet of the hospital, my object being in a great measure to determine the relation that the liquid taken and discharged bore to each other. He used alkaline medicines, but without any marked effect either upon the quantity or quality of the urine; and the warm bath was tried every other day, for a fortnight, without any advantage. His skin was dry, and he was emaciated; he complained of thirst; his appetite was good, and his bowels regular.

After remaining five weeks in the hospital, finding the urine reduced to its natural quantity, not passing more than three pints daily, he wished to return home, as he said he could perform some light work. His emaciation was stationary.

The patient laboring under Diabetes Insipidus was a tailor by trade, and 34 years of age; he complained of very severe thirst, and passed in twenty-four hours from 14 to 18 pints of watery urine, having no sweetness, and a pint of which by evaporation yielded only about one drachm of saline extractive matter. His appetite was good, his skin was dry, although not wholly unperspiring; he was an out patient of the hospital, and tried various alkaline and acid medicines with preparations of iron without any good effect, he also used the warm bath every other day, and without advantage, for three weeks. At length giving, for some time, and till the gums were slightly affected, six grains of pilul. hydrargyri every night, his thirst gradually abated, the urine was reduced to its natural state and quantity, and he regained his ufual health. In this cafe the liquid taken and urine paffed were in due proportion to each other.

From what I have feen of Diabetes Mellitus, I am led to think that a reduction of the urine taking place from diminifhing the liquid taken, has induced patients, not unfrequently, to think themfelves getting well, although the diabetic macies remains unaltered; and in the fame manner, in many inftances, it feems probable, that in treating Diabetes, authors have afcribed to the quality of the liquid taken an effect that fhould more probably be given to a diminution of its quantity. I make this remark, finding that an intelligent correfpondent has, in the laft number of the Medical Journal, given fome cafes of this complaint, in which lime water feems to have effected a cure. In thefe cafes no mention being made of the quantity of liquid taken daily, it is probable, while ufing the lime-water, the fum of the whole liquid taken might be confiderably reduced. In remarking upon thefe cafes, I fhould regret much to appear to doubt either the fidelity or the judgment of your correfpondent; but having feen the fatality of this difeafe, in feveral inftances, and knowing, from an able communication which I have lately received from Mr. Reeve, one of the Prefidents of the Royal Medical Society at Edinburgh for the prefent year, that its fatality has been extenfively evinced in the Edinburgh Infirmary, and under the ableft treatment, (for it appears, that of thirteen diabetic patients admitted into that Infirmary in no very long time, twelve have died) I was furprifed to find it fo readily yielding to fo fimple a medicine as lime-water. I may alfo obferve, that the event of the cafe of the nobleman mentioned by Willis does not appear; his complaint was only relieved at times, and perhaps as much from the diminifhed quantity of liquid taken, as from its quality, whether lime-water or any other liquid. Willis acknowledges that this patient's complaint frequently returned.

Before I conclude, I wifh to guard againft a fource of error in comparing the liquid taken and difcharged in Diabetes. In all the cafes which I have feen, I fhould have concluded, that more urine was paffed than there was liquid taken, had I been fatisfied with comparing the liquid ingefta and egefta, for the firft twenty-four hours, or even two or three days, after they fell under my care; for I have conftantly found, for a fhort time, the urine furpaffing the limited quantity of liquid taken; but this excefs I have always, and I think with reafon, attributed to the greater quantity of liquid taken by patients, for the laft twenty-four hours, or perhaps two or three days, previous to their adopting any mode of treatment. Thus, in the firft cafe of Diabetes (if it may be fo called) mentioned in the laft Medical Journal, when it is faid that the the patient paffed ten pints of urine in one night, although he took only one pint of liquid, I fhould contend, that this occurrence is no proof that the urine exceeds the liquid taken; for before fuch conclufion is warranted, it muft be evident, that it fhould be afcertained what liquid had been taken by the patient during the previous twenty-four or perhaps thirty-fix hours.

In making thefe remarks, I do not mean to deny that there are cafes in which the urine exceeds the liquid taken, for my ingenious correfpondent above-mentioned has remarked, that fuch feems to have been the cafe with fome patients, lately, in the Edinburgh Infirmary.

---

### *To the Editors of the Medical and Phyfical Journal.*

Gentlemen,

